# FDA-Approved Amoxapine Effectively Promotes Macrophage Control of Mycobacteria by Inducing Autophagy

**DOI:** 10.1128/spectrum.02509-22

**Published:** 2022-09-21

**Authors:** Jia Wang, Jian Sha, Emily Strong, Ashok K. Chopra, Sunhee Lee

**Affiliations:** a Department of Microbiology and Immunology, University of Texas Medical Branch, Galveston, Texas, USA; PHRI-Rutgers

**Keywords:** *Mycobacterium tuberculosis*, autophagy, host-directed therapeutics, antidepressant, mTOR, macrophages

## Abstract

Antibiotic resistance poses a significant hurdle in combating global public health crises, prompting the development of novel therapeutics. Strategies to enhance the intracellular killing of mycobacteria by targeting host defense mechanisms offer numerous beneficial effects, which include reducing cytotoxicity caused by current lengthy anti-tubercular treatment regimens and slowing or circumventing the development of multidrug-resistant strains. The intracellular pathogen Mycobacterium tuberculosis infects macrophages and exploits host machinery to survive and multiply. Using a cell-based screen of FDA-approved drugs, we identified an antidepressant, Amoxapine, capable of inhibiting macrophage cytotoxicity during mycobacterial infection. Notably, this reduced cytotoxicity was related to the enhanced intracellular killing of Mycobacterium bovis BCG and M. tuberculosis within human and murine macrophages. Interestingly, we discovered that postinfection treatment with Amoxapine inhibited mTOR (mammalian target of rapamycin) activation, resulting in the induction of autophagy without affecting autophagic flux in macrophages. Also, inhibition of autophagy by chemical inhibitor 3-MA or knockdown of an essential component of the autophagic pathway, ATG16L1, significantly diminished Amoxapine’s intracellular killing effects against mycobacteria in the host cells. Finally, we demonstrated that Amoxapine treatment enhanced host defense against M. tuberculosis in mice. In conclusion, our study identified Amoxapine as a novel host-directed drug that enhances the intracellular killing of mycobacteria by induction of autophagy, with concomitant protection of macrophages against death.

**IMPORTANCE** The emergence and spread of multidrug-resistant (MDR) and extensive drug-resistant (XDR) TB urges the development of new therapeutics. One promising approach to combat drug resistance is targeting host factors necessary for the bacteria to survive or replicate while simultaneously minimizing the dosage of traditional agents. Moreover, repurposing FDA-approved drugs presents an attractive avenue for reducing the cost and time associated with new drug development. Using a cell-based screen of FDA-approved host-directed therapies (HDTs), we showed that Amoxapine inhibits macrophage cytotoxicity during mycobacterial infection and enhances the intracellular killing of mycobacteria within macrophages by activating the autophagy pathway, both *in vitro* and *in vivo*. These findings confirm targeted autophagy as an effective strategy for developing new HDT against mycobacteria.

## INTRODUCTION

Tuberculosis (TB), caused by Mycobacterium tuberculosis (*Mtb*) infection, is one of the leading causes of mortality worldwide. It is estimated that in 2020, TB killed 1.5 million people and 10 million new TB cases emerged (1). The emergence and spread of multidrug or extensively drug-resistant (MDR/XDR) strains are problematic in maintaining progress in the global fight against TB. The current recommended treatment for drug-sensitive TB consists of intensive and continuation phases using combinations of four first-line TB drugs, with the regimen lasting 6 to 9 months (2). Drug-resistant TB treatment requires incorporating more second-line TB drugs in both phases and prolonged treatment duration, lasting up to 2 years (3). The escalating evolution rate of drug-resistant TB strains highlights the need for developing alternative treatment strategies.

Host-directed therapies (HDT), aimed at potentiating bactericidal responses, may serve as potential new anti-TB treatments to prevent the occurrence of drug resistance, shorten treatment duration, and reduce immunopathology. As the primary target of *Mtb* infection, macrophages may effectively eliminate intracellular bacteria via complex host defense mechanisms or provide a significant survival and growth niche to *Mtb*, contributing to distinct disease outcomes (4). As an essential cellular homeostatic process by which damaged or obsolete cell contents are targeted for lysosomal degradation to recycle nutrients, autophagy is a vital host defense mechanism against intracellular pathogens, including *Mtb* (5). Activation of autophagy by physiological means such as nutrient starvation or pharmacological treatment with the mTOR (mammalian target of rapamycin) inhibitor rapamycin significantly reduces the intracellular survival of *Mtb* in infected macrophages (6). mTOR, a serine/threonine kinase, is a master regulator of cellular metabolism. In addition to regulating cell growth and proliferation in response to a wide range of cues, mTOR and the signaling pathways it ensues are deregulated in many human diseases (7). mTOR also plays a crucial role in regulating autophagy (8). A genome-wide small interfering RNA (siRNA) screen also identified host factor networks that regulate *Mtb* survival through autophagy activation independent of mycobacterial strains (9). Nevertheless, *Mtb* has evolved sophisticated countermeasures, such as producing virulence mediators, including ESAT-6, Eis, PE_PGRS47, and other PE and PPE proteins to inhibit autophagy (10–14).

Interestingly, as drug repurposing provides a rapid approach to identifying novel therapeutics against *Mtb* and other intracellular/extracellular bacterial pathogens (15–18), previous screening efforts with different FDA-approved drug libraries have identified several HDTs which could restrict intracellular *Mtb* growth. All of these drugs induce autophagy of host cells with no significant bactericidal activity against *Mtb* in axenic culture. These HDTs include the antidepressants nortriptyline and fluoxetine, the antipsychotic prochlorperazine edisylate, two anticonvulsants, carbamazepine, valproic acid, and the epidermal growth factor receptor inhibitor gefitinib (19–21). Carbamazepine effectively controls MDR-TB in a mouse model (21).

In addition, HDTs identified by screens with FDA-approved drugs also show broad-spectrum efficacy against intracellular and extracellular bacterial pathogens (17). Specifically, Andersson et al. (15) have previously identified 58 FDA-approved drugs that effectively inhibited host cell cytotoxicity during Yersinia pestis CO92 infection when used as the postinfection treatment in RAW 264.7 murine macrophages. Among them, three drugs protected mice against infection with various bacterial pathogens, including Y. pestis, Klebsiella pneumoniae, and Clostridium difficile, by targeting the host (15, 16, 22). Based on these observations, we hypothesized that different bacterial pathogens rely on consensus host pathways or functions for survival, and drugs which perturb these host functions are promising treatment candidates. Due to biosafety concerns, Mycobacterium bovis BCG has been one of the most used surrogates of *Mtb* for anti-TB drug screening. Therefore, we performed a cell-based drug screen of these 58 FDA-approved drugs using BCG. We initially screened for drugs which reduced macrophage cell death upon infection with BCG, then examined the effects of the selected drugs on intracellular BCG survival. In this study, we determined that the antidepressant Amoxapine most effectively enhanced the intracellular killing of mycobacteria through the activation of autophagy in macrophages. Importantly, monotherapy with Amoxapine also enhances host defense against *Mtb in vivo*, suggesting that Amoxapine is a potential HDT against *Mtb*.

## RESULTS

### FDA-approved drugs inhibit BCG-induced macrophage cytotoxicity and restrict intracellular BCG survival.

To streamline the screen for host-directed FDA-approved drugs against mycobacterial infection, we first employed a cytotoxicity assay to identify compounds which reduced mycobacterial infection-induced cell cytotoxicity. Among the prioritized drugs, intracellular bacterial survival was evaluated.

**FIG 1:**
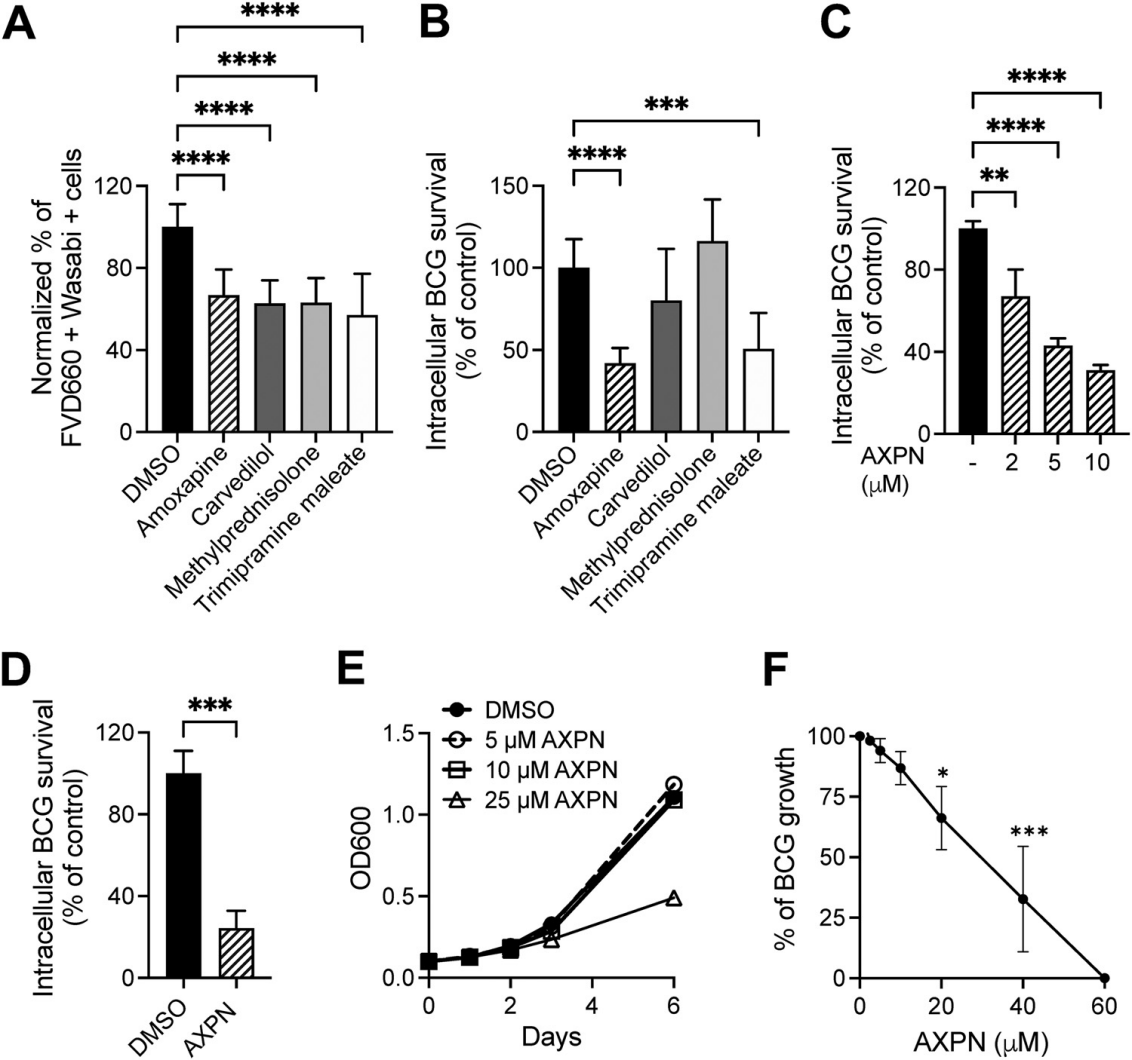
FDA-approved drugs reduce Mycobacterium bovis BCG-induced cytotoxicity and inhibit intracellular BCG survival in macrophages. (A) RAW 264.7 cells were treated with drugs at 10 μM concentration after BCG infection for 3 h, and cytotoxicity was determined by Fixable Viability Dye eFluor 660 staining on day 1 postinfection. Data show the percentage of infected dead cells in drug-treated groups versus the DMSO-treated group. (B) Intracellular BCG survival was determined in RAW 264.7 cells with BCG infection and postinfection treatment with 10 μM drugs on day 2 postinfection. (C) RAW 264.7 cells were infected with BCG at a multiplicity of infection (MOI) of 10 for 3 h and then treated with Amoxapine at the indicated concentrations. Intracellular BCG survival was determined on day 2 postinfection. (D) THP1 cells were treated with 10 μM Amoxapine after 3 h of BCG infection, and intracellular BCG survival was enumerated on day 3 postinfection. (E) To determine the direct bactericidal activity of Amoxapine, BCG growth in 7H9+OADC liquid broth culture was determined by measuring optical density at 600 nm (OD_600_) at different time points with various concentrations of Amoxapine. (F) A resazurin microtiter assay was used to evaluate the sensitivity of BCG to Amoxapine treatment after 5 days of incubation. Data are shown as means ± standard deviations for three independent experiments. One-way analysis of variance (ANOVA) with Dunnett’s multiple-comparison test or Student’s *t* test was used to compare drug-treated groups to the control group. *, *P* < 0.05; **, *P* < 0.01; ***, *P* < 0.001; ****, *P* < 0.0001.

To screen for drugs which reduced the cytotoxicity of RAW 264.7 macrophages when administered after infection with BCG, we used flow cytometry-based live/dead staining, which selectively detects cells undergoing necrosis (23). Drugs were tested at a concentration of 10 μM. Among the 58 drugs tested (Fig. S1), following 24 h postinfection with wasabi-labeled BCG at a multiplicity of infection (MOI) of 10, the percentage of infected cytotoxic cells, shown as BCG-wasabi and fixable viability dye (FVD) 660 double-positive macrophages, was reduced by treatment with four drugs (Fig. 1A). These compounds were Amoxapine, Carvedilol, Methylprednisolone, and Trimipramine maleate (Fig. 1A). Methylprednisolone belongs to the corticosteroids, with a proven beneficial effect on TB patient survival by acting on the host (24). Corticosteroids further inhibit *Mtb-*induced necrotic cell death by abrogating mitochondrial membrane permeability transition without affecting intracellular *Mtb* load (25). Carvedilol is a beta-blocker used to treat hypertension and heart failure. Amoxapine and Trimipramine maleate are antidepressants.

Focusing on the four drugs identified from the screen, we tested their effects on intracellular BCG survival. The drugs were administered postinfection and tested at a concentration of 10 μM in RAW 264.7 cells. At 48 h postinfection with an MOI of 10, two antidepressants, Amoxapine and Trimipramine maleate, significantly inhibited intracellular BCG survival compared to untreated cells (Fig. 1B). The antidepressant Trimipramine maleate was reported to reduce viable intracellular BCG load in a previous study (21). Therefore, we focused on Amoxapine because this drug was also effective against other pathogens, such as Y. pestis, C. difficile and K. pneumoniae in our earlier studies (15, 16, 22).

Next, we confirmed Amoxapine’s ability to inhibit intracellular BCG survival in murine and human macrophages without acting directly on BCG. Post-infection treatment with Amoxapine significantly reduced intracellular BCG survival in a dose-dependent manner in RAW 264.7 cells (Fig. 1C). Treatment with 10 μM Amoxapine alone did not cause cell cytotoxicity in either RAW 264.7 or THP1 cells, measured by 3-(4,5-dimethylthiazol-2-yl)-2,5-diphenyltetrazolium bromide (MTT) assays (Fig. S2A and B). Post-infection treatment with 10 μM Amoxapine markedly inhibited intracellular BCG survival in macrophages, including RAW 264.7 and THP1 cells (Fig. 1C and D), without affecting bacterial growth in liquid broth culture (Fig. 1E). A resazurin microtiter assay demonstrated that MIC_90_ of Amoxapine against BCG was 53 μM (Fig. 1F).

### Amoxapine induces mTOR-dependent autophagy.

**FIG 2:**
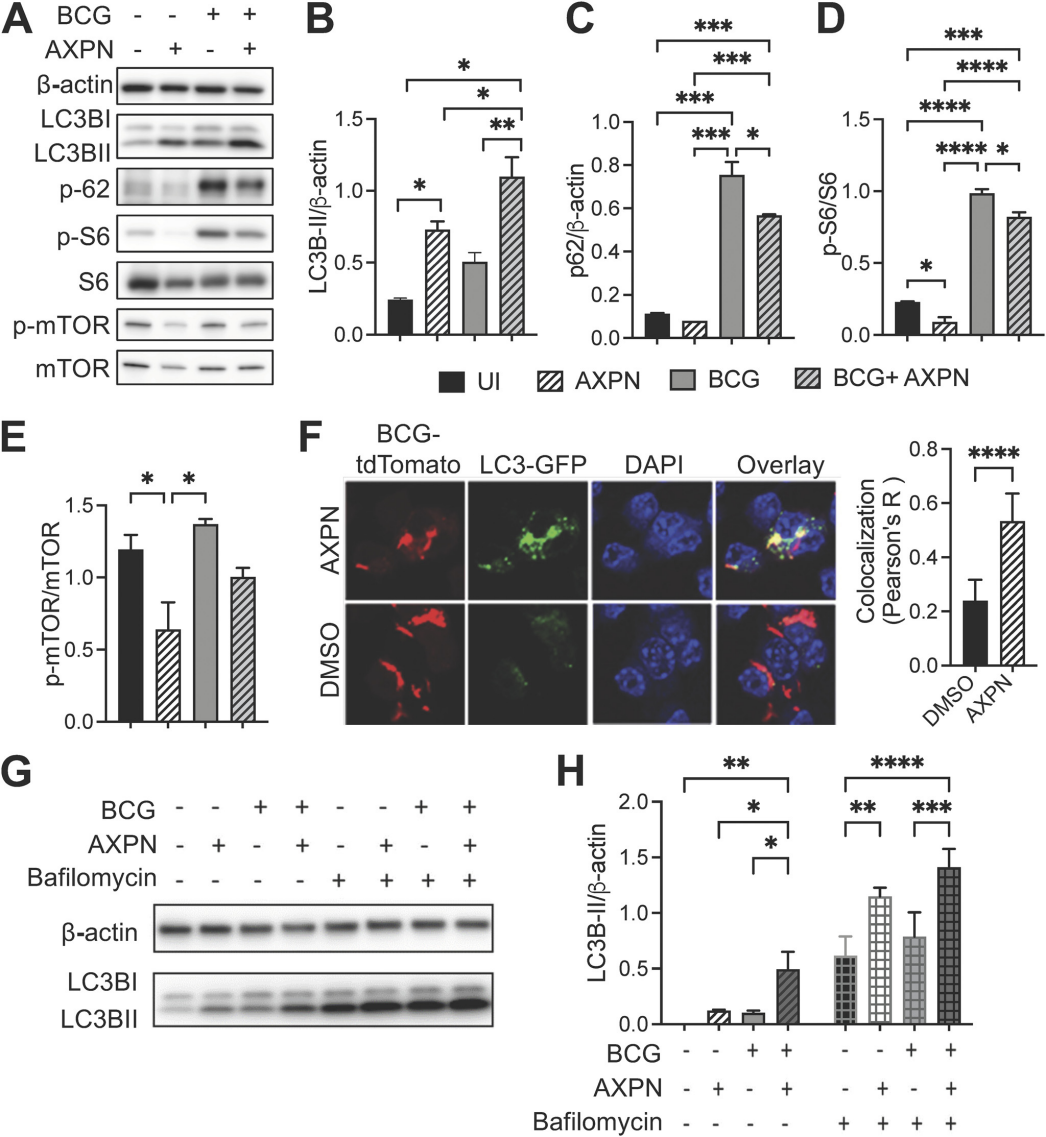
Amoxapine induces autophagy in an mTOR-dependent manner. (A) RAW264.7 cells were infected with BCG at an MOI of 10 for 3 h and then treated with 10 μM Amoxapine for 24 h, and Western blots were used to determine LC3B-II, p62, phosphor S6 (S235/236), total S6, phosphor mTOR (S2448), total mTOR, and actin levels. (B to E) Quantifying protein levels are shown in panel A using Image J. (F) RAW 264.7 LC3-GFP cells were infected with BCG-tdTomato at an MOI of 10 for 3 h and then treated with 10 μM Amoxapine for 24 h. Cells were fixed and stained with DAPI (4′,6-diamidino-2-phenylindole). Confocal images were acquired using an A1 Nikon confocal microscope with a 60× objective, and Pearson’s correlation was used to quantify colocalization between LC3-GFP and BCG-tdTomato. (G to H) Representative Western blots of LC3B-II in RAW 264.7 cells with BCG infection and 10 μM Amoxapine treatment at 24 h postinfection. Bafilomycin A was added to the cells at a concentration of 1 μM for 4 h before protein extraction. Image J was used for quantifying the LC3B-II level. Data represent means ± standard deviations for three independent experiments. One-way ANOVA with Dunnett’s multiple-comparison test or Student’s *t* test was used for statistical analysis. *, *P* < 0.05; **, *P* < 0.01; ****, *P* < 0.0001.

Several previous reports have described the diverse effects of antidepressants on autophagy (26, 27). Autophagy has emerged as a promising host-directed target because it is a protective process that inhibits intracellular *Mtb* growth in host cells (28). Therefore, we hypothesized that Amoxapine might induce autophagy in mycobacteria-infected cells. Autophagy-related proteins (ATGs) are critical players in the process of autophagy. Changes in ATG gene expressions and post-translational protein modifications are often used as markers for autophagy. Because it is a member of the ATG8 protein family, converting soluble microtubule-associated protein 1 light chain LC3-I to autophagosomal membrane-bound LC3-II by phosphatidylethanolamine conjugation is one of the most used markers for autophagy (29). Furthermore, p62, a cargo adaptor protein that targets substrates for autophagosome degradation, also degrades when autophagy is induced and is widely used as a predictor of autophagic flux. At 24 h postinfection with BCG, we observed significantly enhanced levels of LC3B-II protein and reduced levels of p62 in RAW 264.7 cells treated with 10 μM Amoxapine compared to that in their their corresponding vehicle-treated controls (Fig. 2A to C). mTOR regulates cell growth and homeostasis and is usually a negative regulator of autophagy (8). Although different mycobacteria species are potent activators of mTOR, their ability to induce autophagy varies (30, 31). Therefore, we examined the effects of Amoxapine treatment on mTORC1 and its downstream activities. S6 is a component of the 40S ribosomal subunit downstream of mTOR, and phosphorylation of S6 upregulates protein translation (32). After 24 h of treatment, we observed that Amoxapine significantly reduced phosphorylation (Ser235/236) of ribosomal S6 with or without BCG infection (Fig. 2A and D). The ratio of phosphorylation at Ser 2448 of mTOR to total mTOR was also significantly reduced in Amoxapine-treated cells compared to that in vehicle-treated uninfected cells. A similar trend was observed in BCG-infected cells, although the difference was not statistically significant (Fig. 2A and E). These data indicated that Amoxapine inhibited mTOR signaling activation in macrophages, resulting in significant autophagy induction.

Next, we used confocal imaging to evaluate whether Amoxapine-induced autophagy could target intracellular BCG. RAW264.7 cells with stable expression of LC3-GFP were infected with tdTomato-labeled BCG and then treated with Amoxapine or vehicle control dimethyl sulfoxide (DMSO) for 24 h. Our results showed that Amoxapine treatment significantly increased the colocalization between LC3 and intracellular BCG compared to that in the DMSO-treated group (Fig. 2F). During autophagy, cytoplasmic LC3 is processed and recruited to the autophagosomal membranes; therefore, cells undergoing autophagy can be identified by visualizing fluorescently labeled LC3 puncta. LC3-GFP puncta formation was also significantly upregulated in cells infected with BCG and treated with 10 μM Amoxapine compared to that in infected cells without the drug treatment (Fig. 2F and Fig. S3). Notably, treating cells with the autophagosome-lysosomal fusion inhibitor bafilomycin A1 further increased LC3-II levels in cells treated with Amoxapine compared to uninfected and BCG-infected macrophages (Fig. 2G and H). These results confirmed that Amoxapine significantly enhanced autophagosome formation and maintained the autophagic flux.

### Amoxapine stimulates the autophagic killing of mycobacteria.

**FIG 3:**
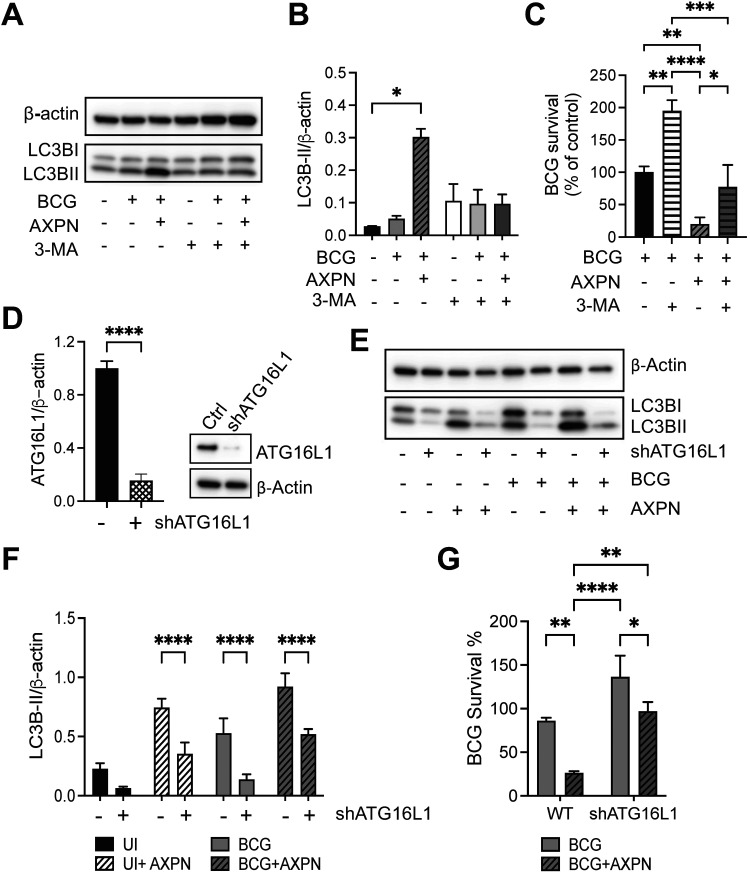
Autophagy contributes to Amoxapine-mediated suppression of intracellular survival of BCG in macrophages. (A) RAW 264.7 cells were infected with BCG for 3 h and treated with 10 μM Amoxapine for 24 h in the presence or absence of 5 mM 3-MA. Western blots detected LC3B-II and actin levels. (B) Image J was used to quantify protein expression levels. (C) Intracellular BCG survival was enumerated by CFU in RAW 264.7 with BCG infection and 10 μM Amoxapine treatment in the presence or absence of 3-MA at day 2 postinfection. (D) ATG16L1 expression levels were detected by Western blots in control and shATG16L1 knockdown of RAW264.7 cells. (E to F) Western blots and quantification of LC3B-II levels in control and shATG16L1 knockdown of RAW264.7 cells with BCG infection and treatment with 10 μM Amoxapine at 24 h postinfection. (G) Intracellular survival of BCG was enumerated by CFU in control and shATG16L1 knockdown of RAW264.7 cells with BCG infection and 10 μM Amoxapine treatment at day 1 postinfection. Data represent means ± standard deviations for three independent experiments. One-way ANOVA with Dunnett’s multiple-comparison test was used for statistical analysis to compare the 3-MA-treated group to the untreated group or ATG16L1 knockdown groups to the control group. *, *P* < 0.05; **, *P* < 0.01; ****, *P* < 0.0001.

To further investigate whether Amoxapine treatment effectively controls intracellular mycobacterial survival through autophagy, we compared intracellular BCG survival between Amoxapine-treated cells in the presence or absence of an autophagy inhibitor, 3-methyladenine (3-MA). In line with previous findings, treatment with Amoxapine-induced autophagy was evidenced by increased LC3B-II levels (Fig. 3A and B). Treatment with 3-MA abolished the induction of LC3B-II triggered by Amoxapine treatment. While Amoxapine significantly reduced the intracellular bacterial load of BCG compared to that in infected cells without 3-MA treatment, intracellular BCG survival was significantly increased with 3-MA in cells treated with Amoxapine (Fig. 3C).

To confirm the role of autophagy in suppressing intracellular mycobacterial growth, ATG16L1, a component of the ATG12-ATG5-ATG16L1 conjugation system essential for elongation and expansion of autophagosome, was knocked down using short hairpin RNA (shRNA) lentiviral transduction in RAW 264.7 cells (Fig. 3D). Knockdown of ATG16L1 significantly diminished LC3B-II levels under all conditions, including untreated, BCG-infected, Amoxapine-treated, BCG-infected, and Amoxapine-treated cells (Fig. 3E and F). In line with previous results, at 24 h postinfection, Amoxapine treatment significantly suppressed intracellular BCG survival in control cells. Knockdown of shATG16L1 diminished the effects of Amoxapine on suppressing intracellular BCG survival (Fig. 3G). Thus, these results suggest that autophagy plays a significant role in intracellular killing of mycobacteria triggered by Amoxapine treatment.

### Post-infection treatment with Amoxapine restricts intracellular *Mtb* survival.

**FIG 4:**
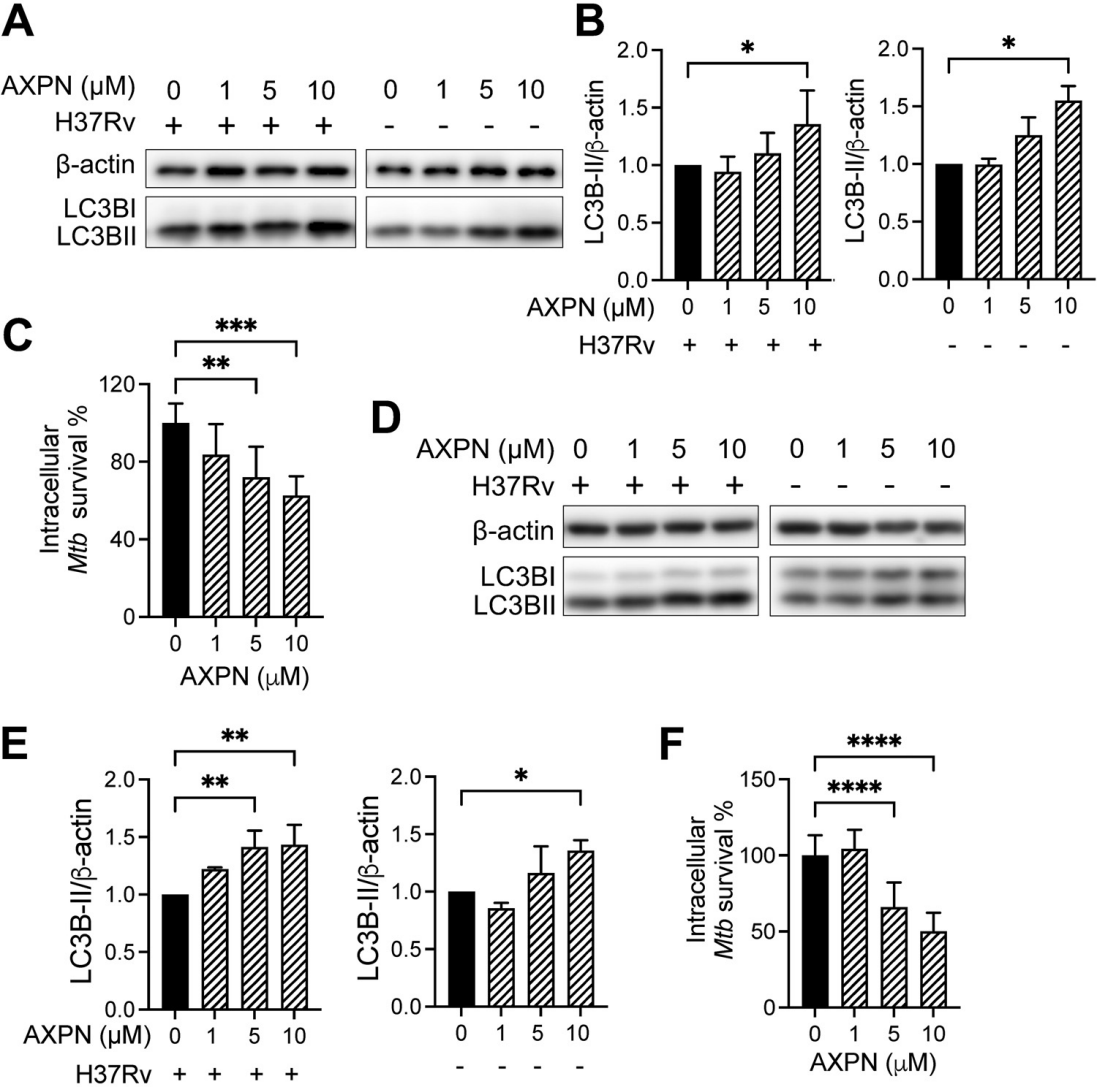
Amoxapine suppresses intracellular Mycobacterium tuberculosis (*Mtb*) survival in macrophages. (A to C) Murine bone marrow-derived macrophages (BMDMs) were infected with *Mtb* H37Rv at an MOI of 10 for 4 h and treated with Amoxapine at indicated concentrations, or they were uninfected and treated with Amoxapine for 3 days. (A and B) LC3B-II and actin levels in murine BMDMs were determined by Western blots and quantified by Image J. (C) Intracellular survival of *Mtb* H37Rv was determined by CFU enumeration. (D and E) THP1 cells were infected with *Mtb* H37Rv at an MOI of 10 for 4 h and then treated with Amoxapine at indicated concentrations or uninfected and treated with Amoxapine for 3 days. LC3B-II levels were determined by Western blots and quantified by Image J. (F) Intracellular survival of *Mtb* H37Rv in THP1 cells was determined by CFU enumeration. Data represent mean ± standard deviations for three independent experiments. One-way ANOVA with Dunnett’s multiple-comparison test or Student’s *t* test was used to compare drug-treated groups to the control group. *, *P* < 0.05; **, *P* < 0.01; ***, *P* < 0.001; ****, *P* < 0.0001.

We further tested the host-directed anti-mycobacterial abilities of Amoxapine on *Mtb* in murine and human macrophage cell lines at different concentrations. At postinfection treatment concentrations of up to 10 μM and under no-infection conditions, Amoxapine increased LC3B-II levels in primary murine bone marrow-derived macrophages and THP1 cells at 72 h postinfection (Fig. 4A, B, D, to E). LC3B-I levels in primary murine bone marrow-derived macrophages were relatively low but visible over a longer exposure time (Fig. S4A). In these cells, Amoxapine substantially decreased intracellular survival of *Mtb* H37Rv at 5- and 10-μM concentrations at 72 h postinfection (Fig. 4C and F). We also tested the effects of two primary metabolites of Amoxapine, 7-hydroxyamoxpine and 8-hydroxyamoxapine, on intracellular *Mtb* survival in THP1 cells. Our results showed that both metabolites significantly enhanced autophagy at 10 μM concentration and reduced *Mtb* survival (Fig. S5).

### Amoxapine is effective in treating *Mtb in vivo*.

**FIG 5:**
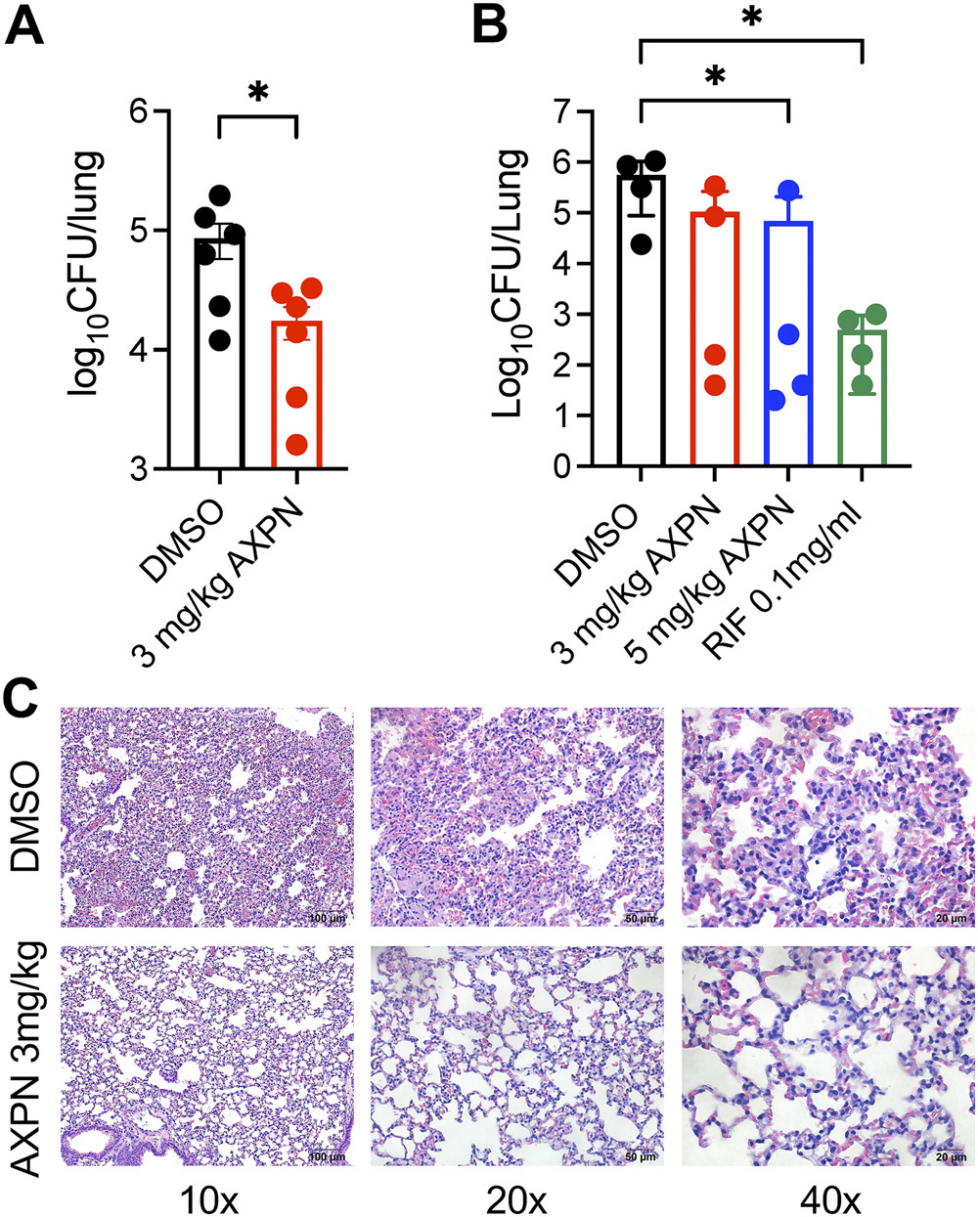
Amoxapine stimulates the killing of *Mtb in vivo*. *Mtb* burden in the lungs of BALB/c mice (A) was enumerated at 2 weeks *ad libitum* treatment with Amoxapine or DMSO after intranasal infection with ~100 CFU H37Rv. A nonparametric unpaired *t* test was used to determine significant differences between the Amoxapine-treated and control DMSO groups. (B) *Mtb* burden in the lungs of C57BL/6 mice was enumerated at 2 weeks *ad libitum* treatment with Amoxapine or DMSO after intranasal infection with ~860 CFU H37Rv. A nonparametric (Kruskal-Wallis) test with Dunn’s post hoc test was used to compare drug-treated groups to the control DMSO group. (C) Representative hematoxylin and eosin (H&E)-stained lung sections at 2 weeks post-treatment with Amoxapine or vehicle control DMSO, as described in panel A. *, *P* < 0.05.

To determine whether Amoxapine is effective against *Mtb in vivo*, we used two mice models, BALB/c and C57BL/6, to confirm its efficacy. We intranasally infected BALB/c mice with a low dose of approximately 100 CFU of *Mtb* strain H37Rv to test the efficacy of 3 mg/kg of Amoxapine *in vivo*. The infected animals were left untreated for 2 weeks to facilitate disease progression and then treated with Amoxapine for 2 weeks. For low-dose *Mtb* infection, a 2-week *ad libitum* treatment with 3 mg/kg Amoxapine significantly reduced bacterial burdens in the lungs compared to the vehicle DMSO-treated group (Fig. 5A). Similarly, we infected C57BL/6 mice with a high dose of ~860 CFU of *Mtb* strain H37Rv by intranasal inoculation, since C57BL/6 mice are more resistant to *Mtb* infection than BALB/c mice after intranasal inoculation. When treated with 3 or 5 mg/kg Amoxapine *ad libitum* for 2 weeks, *Mtb*-infected C57BL/6 mice demonstrated a substantial reduction of bacterial burden in the lungs (Fig. 5B). Treatment with first-line anti-mycobacterial antibiotic rifampicin (33), as expected, had a significant effect on *Mtb* burden (Fig. 5B). Diminished leukocyte accumulation, inflammatory pulmonary infiltrates, and decreased lung lesions were observed in Amoxapine-treated lung tissues compared to those in the control group in low-dose infection (Fig. 5C). These results indicated that Amoxapine enhanced host defense against *Mtb in vivo*.

In conclusion, Amoxapine induces autophagy by mTOR inhibition, leading to enhanced intracellular bacterial clearance. Amoxapine also showed efficacy for reducing *Mtb* loads *in vivo* after 2 weeks of treatment. Our study demonstrated that repurposing FDA-approved drugs can provide an efficient alternative to streamlining screens for novel host-directed therapeutics against mycobacterial infection.

## DISCUSSION

Our initial cell-based screen with M. bovis BCG took a different approach from previously performed screens by others. Most previous mycobacterial cell-based screening efforts used intracellular bacterial growth as the sole screening criteria (19) or measured complex visual outputs, including host cell morphology, toxicity, bacterial number, and intensity (20). Instead, we conducted a focused, small-sized screen of 58 host-directed drug hits (Table S1) identified from a previous drug screen against the facultative intracellular pathogen Y. pestis. We first utilized a cell viability assay to determine drug candidates that inhibited BCG-infected macrophage cell death (Fig. S1). The prioritized drugs were then evaluated by their activities against intracellular bacterial survival and extracellular growth. Although further experiments are warranted to confirm their significance against *Mtb* infection in more clinically relevant cells, such as primary human macrophages or mouse lung cells, our simple yet efficient screening strategy identified 2 drugs, namely, Methylprednisolone and Trimipramine maleate (Fig. 1A), which had been previously reported to be effective against *Mtb* (21, 25), thus increasing the creditability of our screening method. It has been demonstrated that corticosteroids such as Methylprednisolone inhibit *Mtb-*induced necrotic host cell death by abrogating mitochondrial membrane permeability transition without affecting intracellular *Mtb* survival (25). Meanwhile, Trimipramine maleate, an antidepressant, reduced intracellular BCG survival by activating mTOR-independent autophagy (21). A previous study has shown that the antidepressant Desipramine increases macrophage bactericidal activity against M. marinum only in zebrafish with high levels of leukotriene A4 hydrolase, but not in the wild-type fish (34). Desipramine prevents high tumor necrosis factor-mediated macrophage necrosis by inhibiting ceramide production (34). Our data showed that Amoxapine also inhibited macrophage necrosis without affecting apoptosis (Fig. S6).

Furthermore, our current study has demonstrated for the first time that another antidepressant drug, Amoxapine, could inhibit death of mycobacteria-infected cells and suppress intracellular growth of BCG and *Mtb*. The primary metabolites of Amoxapine, 7-hydroxyamoxapine and 8-hydroxyamoxapine, also demonstrated enhanced activities against intracellular *Mtb* individually. The combination of these two metabolites may yield stronger inhibition activity than the individual drug, which remains to be determined. We found that Amoxapine had no significant anti-mycobacterial activity against BCG and *Mtb* at 10 μM concentration (Fig. 1E and Fig. S4B), yet notably inhibited intracellular growth of BCG and *Mtb*. However, it started to exhibit killing of extracellular mycobacteria at an MIC_90_ of 53 μM for BCG and an MIC_90_ of 67 μM for *Mtb* (Fig. 1F and Fig. S4B). This observation is in line with a previous host-directed drug screen against Coxiella burnetii, in which many of the 75 host-directed drugs also decreased the growth of *C. burnetii* in an axenic medium when tested at higher concentrations (17). Although the exact concentrations of Amoxapine within macrophages were not known when cells were treated with 10 μM Amoxapine *in vitro*, it is possible that Amoxapine’s direct antimycobacterial activity may work together with its effects on inducing host autophagy to control intracellular mycobacterial survival. These findings are distinct from those of previous reports in which Amoxapine provided protection against Y. pestis CO92, K. pneumoniae, and C. difficile solely through host-directed mechanisms. In these studies, Amoxapine displayed no direct antimicrobial effects against these bacteria, with MICs determined to be above 100 μg/mL (318 μM) (15, 16, 22).

How Amoxapine precisely inhibits mycobacterial growth at higher concentrations remains unknown. It has been reported that the antidepressant Loxapine, which structurally relates to and can be metabolized to Amoxapine, suppresses intracellular multiple antibiotic-resistant Salmonella Typhimurium within macrophages, possibly through inhibiting the efflux pump activity of bacteria (35). Another antidepressant, Imipramine, inhibits mycobacterial topoisomerase I activity by targeting the enzyme’s metal-binding pocket and affects mycobacterial growth (36). Therefore, further studies will be required to elucidate the molecular mechanisms by which Amoxapine affects extracellular BCG growth at high concentrations.

Autophagy is a vital cell defense mechanism that sequesters intracellular mycobacteria into autophagosome organelles and targets lysosomal degradation. We observed that postinfection treatment with Amoxapine significantly increased LC3B-II and autophagosome formation in mycobacteria-infected macrophages (Fig. 2). Furthermore, Amoxapine induced autophagic processes without affecting autophagic flux. Inhibition of autophagy by a chemical inhibitor or a genetic approach diminished Amoxapine’s effects on intracellular bacterial killing (Fig. 3). We demonstrated that Amoxapine significantly inhibited a negative autophagy regulator, mTOR, and its downstream signaling, resulting in induced autophagy (Fig. 2A to E). How Amoxapine modulates mTOR signaling remains elusive. Many tricyclic antidepressants are functional inhibitors of the acid sphingomyelinase, although no reports of Amoxapine functioning through this enzyme have been found yet. The acid sphingomyelinase is an endo-lysosomal protein which hydrolyzes sphingomyelin to phosphorylcholine and ceramide in the lysosome (37). Inhibition of baseline acid sphingomyelinase activity by the antidepressant Imipramine significantly increased autophagy while preserving autophagic flux by disrupting lysosomal nutrient-sensing complex (LYNUS) signaling, including mTOR (38). Further investigations are needed to examine the effects of Amoxapine on acid sphingomyelinase activity to elucidate the molecular mechanisms by which Amoxapine inhibits mTOR signaling.

Interestingly, Amoxapine was also protective against fatal Y. pestis, C. difficile, or K. pneumoniae infection in mice (15, 16, 22). It was further demonstrated that Amoxapine’s protection against lethal C. difficile infection depends on the recruitment of neutrophils to promote IL-33 production (22). IL-33 is a new member of the IL-1 family predominantly expressed by stromal cells, such as epithelial and endothelial cells (39). Following cell injury, IL-33 is released as an alarmin cytokine and binds to the ST2L receptor present on various immune cells to regulate their immune functions (39). IL-33 has also been a promising adjuvant that increases TB-specific cell-mediated response elicited by an *Mtb* antigen 85B DNA vaccine in mice (40). Systematic IL-33 treatment effectively ameliorates *Mtb* infection *in vivo* (41). Given that our *in vitro* infection model was carried out in macrophages, IL-33 was not detected in cells infected with mycobacteria with or without Amoxapine treatment, but was induced by lipopolysaccharide treatment (data not shown). Interestingly, IL-33 protects against haptenizing molecule 2,4,6-trinitrobenzene sulfonic acid-induced colitis in mice by enhancing autophagy through the TLR4-dependent signaling pathway (42). Therefore, it remains to be determined whether Amoxapine can induce IL-33 in *Mtb* infection *in vivo* or whether autophagy is involved in IL-33-mediated protection against other bacterial infections.

As an autophagy inducer, the mTOR inhibitor rapamycin and its analogs have been studied for treating TB. Rapamycin has immunosuppressant activities, variable oral absorption, and metabolism by CYP3A4, which have limited its use in treating TB. Rapamycin has been tested in combination with an anti-TB drug regimen in mouse models and shown detrimental effects when added to the bedaquiline-pretomanid-linezolid regimen, significantly reduced survival in C3HeB/FeJ mice, and increased relapse after treatment in both C3HeB/FeJ and BALB/c mice (43). Our results showed that 2 weeks of *ad libitum* treatment with Amoxapine reduced *Mtb* load in the lungs. Doses of 3 and 5 mg/kg Amoxapine in mice are equivalent to doses of 14.58 and 24.3 mg/day for a 60-kg human, respectively (44), far below the beginning treatment dose of 150 mg/day, with a maximum of up to 600 mg/day, in humans for the antidepressant indication (45). Although Amoxapine delivery routes and doses will be further optimized *in vivo* to improve treatment outcomes, our results showed that Amoxapine did reduce inflammation in the lungs by hematoxylin and eosin (H&E) staining. Our future studies will focus on optimizing the drug-dose regimen for maximum protective efficacy. In addition, the possibility of using Amoxapine as an adjunct therapy for TB will need to be further examined by studying pharmacokinetics and pharmacodynamics parameters and by testing in other pre-clinical animal models of *Mtb* infection.

In summary, our study has demonstrated that repurposing FDA-approved drugs presents an accelerated approach to identifying host-directed drugs against mycobacterial infection. Our findings indicate that the antidepressant Amoxapine inhibits intracellular mycobacterial survival by inducing autophagy through the mTOR-dependent pathway. Amoxapine’s *in vivo* effects against mycobacterial infection further substantiate it as an HDT candidate for TB treatment.

## MATERIALS AND METHODS

### Drugs and reagents.

Fifty-eight FDA-approved drugs were provided as 10 mM stock solutions in dimethyl sulfoxide from the Screen-Well FDA-approved drug library V2 (Enzo life Sciences, BML-2843-0100, Albany, NY) (Table S1). Due to the limited drug stock within the library, Amoxapine was purchased from Sigma-Aldrich (St. Louis, MO) as a dry powder, dissolved in DMSO at a 10 mM stock concentration, and used in the following *in vitro* and *in vivo* validation experiments. The chemical inhibitor 3-methyladenine was purchased from Sigma-Aldrich. Bafilomycin A1 (cat no. 54645) was purchased from Cell Signaling Technologies (Danvers, MA). 7-Hydroxyamoxapine and 8-hydroxyamoxapine were purchased from Santa Cruz Biotechnology (Dallas, TX). An MTT assay kit was purchased from Abcam (Cambridge, MA). A GFP (Green Fluorescent Protein)-Certified Apoptosis/Necrosis detection kit was purchased from Enzo (New York, NY).

### Bacterial strains and culture.

M. bovis (Bacillus Calmette-Guérin) BCG Danish was obtained from William Jacobs’ laboratory (Albert Einstein College of Medicine, The Bronx, NY). M. tuberculosis H37Rv (cat no. 25618) was purchased from American Type Culture Collection (ATCC, Manassas, VA). BCG wasabi and tdTomato were generated by transforming BCG with pTEC-15 wasabi or pTEC27-tdTomato plasmids containing Hygromycin selection marker (gift from David Tobin, Duke University, Durham, NC). All mycobacteria were cultured in Middlebrook 7H9 broth (BD Difco, Franklin Lakes, NJ) or 7H10 agar (BD Difco) supplemented with 0.5% glycerol (Sigma-Aldrich), 0.05% tyloxapol (Sigma-Aldrich), and 10% of oleic acid/albumin/dextrose/catalase supplement (OADC) (BD Diagnostics, Franklin Lakes, NJ). The cultures were grown at 37°C until reaching the log phase. The cultures were washed twice with phosphate-buffered saline (PBS) containing 0.05% tyloxapol, filtered, and adjusted to 2× 10^8^ CFU per mL stock in PBS containing 15% glycerol solution. Aliquots of the cultures were stored at −80°C until use.

### Cell cultures and macrophage infection.

RAW 264.7 murine macrophage cell line and human monocytic cell line (THP1) were purchased from ATCC. RAW 264.7 and RAW LC3-GFP (with microtubule-associated light chain protein [LC3] fused to GFP) cells (31) were grown in high glucose Dulbecco’s Modified Eagle’s Medium (Sigma-Aldrich) supplemented with 10% heat-inactivated fetal bovine serum (FBS, HyClone, Logan, UT) and nonessential amino acids (Sigma-Aldrich). THP1 cells were cultured in RPMI 1640 (Sigma-Aldrich) supplemented with 10% FBS. Bone marrow-derived macrophages (BMDM) were generated by flushing bone marrow cells from femurs and tibias from C57BL/6J mice. Cells were cultured in RPMI 1640 supplemented with 15% L929 conditioned medium, 100 U/mL penicillin, and 100 μg/mL streptomycin for 6 days before use and cultured at 37°C in a 5% CO_2_ incubator. On day 6, adherent cells were collected by detachment using PBS at 4°C for 30 min and seeded in RPMI 1640 supplemented with 15% L929 conditioned medium. THP1 cells were differentiated with 100 ng/mL of phorbol myristate acetate (Sigma-Aldrich) 24 h before infection, and cells were rested for 4 h before infection.

ATG16L1 (autophagy-related 16 like 1) knockdown and control cells were generated by shRNA lentiviral transduction. TRC lentiviral pLKO.1 empty vector control or ATG16L1 shRNA constructs combined (clone IDs: TRCN0000173438, ATCCTGTTCAACAGGTAGAGG; TRCN0000175121, AAAGTTCTCAAGAAAGCTACG; TRCN0000175371, AACAGACTTTGGAAAGATGGG; TRCN0000175562, AAACAATGTCATTGCAGCTGG; TRCN0000176385, AAGTTCTCAAGAAAGCTACGG) (Horizon Discovery, Waterbeach, United Kingdom) were transfected with lentiviral packaging plasmids into HEK293 cells using Viromer Red (OriGene, Rockville, MD). At 48 h posttransfection, lentivirus in the supernatant was collected. RAW 264.7 cells were infected with collected lentivirus in the presence of 10 μg/mL Polybrene. Transduced cells were selected at 48 h postinfection with 10 μg/mL puromycin. Clonal cells were isolated and used for the study.

For mycobacterial infection for the initial screen, host cells were infected with mycobacteria for 3 h at 37°C in a 5% CO_2_ incubator at an MOI of 10. Cells were washed 3 times with PBS and then incubated with a complete medium containing 50 μg/mL gentamicin (Sigma-Aldrich) for 1 h at 37°C. The cell medium was then changed to the complete medium containing 20 μg/mL of gentamicin with drugs or DMSO as a negative control at indicated concentrations. In follow-up intracellular mycobacterial survival experiments, after 1 h of incubation with 50 μg/mL gentamicin, cells were washed three times with PBS, and a cell medium containing Amoxapine or DMSO was used to treat cells without gentamicin. The FDA-approved drugs at the indicated concentrations were added after phagocytosis to identify compounds which restricted intracellular *Mtb* growth rather than inhibiting uptake.

### Cell viability assay.

RAW 264.7 cells were infected with BCG wasabi at an MOI of 10 for 3 h. After three washes with PBS and incubation with 50 μg/mL gentamicin to eliminate extracellular bacteria, macrophages were incubated with 20 μg/mL gentamicin and the indicated drugs for 24 h. Cells were trypsinized and stained with Fixable Viability Dye eFluor 660 (Thermo Fisher Scientific, Waltham, MA) at room temperature for 15 min. Cells were then washed and fixed with Paraformaldehyde Fixative Solution (Thermo Fisher Scientific), and 10,000 cell events were collected using Accuri C6 (BD, Franklin Lakes, NJ) and analyzed using FlowJo software (BD).

### CFU assay.

Mycobacterium-infected macrophages were lysed with radioimmunoprecipitation assay (RIPA) buffer (Sigma-Aldrich) for 10 min at room temperature and then serially diluted in PBS (HyClone). The diluted samples were then plated on Middlebrook 7H10 agar supplemented with 10% OADC and incubated at 37°C for 3 to 5 weeks, then CFU were counted for BCG and M. tuberculosis H37Rv.

### Broth-grown bacteria and sensitivity of BCG or *Mtb* to Amoxapine.

BCG Danish or *Mtb* H37Rv was grown in 7H9 medium containing 10% OADC and 0.05% glycerol to mid-exponential phase with an optical density at 600 nm (OD_600_) of 0.4 to 0.8. The bacterial culture was then diluted to 20-mL subcultures containing either DMSO (negative control) or 10 μM Amoxapine at an OD_600_ of 0.1. The cultures were incubated in a shaker at 37°C, and OD_600_ was measured at the indicated time points.

A resazurin reduction microplate assay was performed to determine the MIC_90_ of Amoxapine against BCG Danish and *Mtb* H37Rv. BCG Danish and *Mtb* H37Rv were grown to mid-exponential phase (OD_600_ = 0.4 to 0.8) and then was diluted to an OD_600_ of 0.002 (4 × 10^5^ CFU/mL) in 7H9S medium (7H9 base medium, 0.05% glycerol, 10% ADC, 1% tryptone, and 0.05% Tween 80). Amoxapine was serially diluted in 7H9 medium in 2-fold dilutions in 96-well plates. The bacteria were incubated with serially diluted Amoxapine for 5 days at 37°C. Next, 30 μL of 0.02% resazurin and 12.5 μL of 20% Tween 80 was added to the wells, and the plate was incubated for a further 24 h for color development. Fluorescence was read with an excitation wavelength of 530 nm and an emission wavelength of 590 nm using a BioTek Synergy H1 plate reader.

### Western blot assay.

Macrophages were lysed with RIPA buffer (Sigma-Aldrich) and precleared by centrifuging at 15,000 rpm for 15 min. A bicinchoninic acid protein assay (Thermo Fisher Scientific) measured total protein concentrations from the supernatants. Equal amounts of proteins were separated by 4% to 20% SDS-PAGE gels (Bio-Rad, Hercules, CA) and then transferred to polyvinylidene difluoride membranes (Millipore, Burlington, MA). After blocking, anti-LC3B (cat no. 2775), anti-phospho S6 (Ser235/236; no. 4857), anti-S6 (no. 2217), anti-phospho mTOR (S2448; no. 2971), anti-mTOR (no. 2983), anti-ATG16L1 (no. 8089), anti-SQSTM1/p62 (no. 5114), and anti-β-actin (no. 4970), from Cell Signaling Technology (Danvers, MA) were used as primary antibodies. Anti-rabbit IgG HRP-linked antibody was used as a secondary antibody (Cell Signaling Technologies, cat no.7074), and Clarity Western ECL Substrate (Bio-Rad) was used to visualize protein bands. Images were acquired using an Amersham Imager 680 (GE Healthcare Life Sciences, Marlborough, MA) and analyzed using ImageJ (NIH).

### Confocal microscopy.

RAW LC3-GFP cells were seeded on a sterilized glass coverslip 1 day before infection. Cells were infected with tdTomato-labeled BCG Danish or unlabeled BCG Danish (MOI = 10), then treated with 10 μM Amoxapine at the indicated concentrations for 24 h. Cells were rinsed once with PBS and fixed in 4% formalin/PBS for 10 min at room temperature. Cells were then washed three times with PBS, and the coverslips were mounted on slides with a prolonged gold antifade reagent and DAPI (4′,6-diamidino-2-phenylindole; Cell Signaling Technologies). Images were taken using an A1 Nikon confocal microscope (Nikon, Tokyo, Japan) and analyzed with NIS Elements (Nikon).

### Mouse *in vivo* infection.

All animal experiments were performed following protocols approved by the Institutional Animal Care and Use Committee of the University of Texas Medical Branch. Female BALB/c or C57BL/6 mice (6 to 8 weeks old) were obtained from the Jackson laboratory. Mice were infected with ~100 or 860 CFU H37Rv via intranasal inoculation. The infection dose was confirmed on day 1 postinfection in the lungs of take-down mice. At 2 weeks postinfection, Amoxapine (3 mg/kg or 5 mg/kg) or solvent control DMSO were administered *ad libitum* in drinking water which was changed every day for 2 weeks. To enumerate bacterial burdens, the lungs of infected animals were aseptically collected in PBS and homogenized using a probe homogenizer. The homogenates were serial diluted and plated on 7H10 agar plates containing OADC. Colonies were counted after incubating the plates at 37°C for 3 to 5 weeks. Lung tissue sections were also fixed in 10% neutral buffered formalin and stained with H&E.

### Statistical analysis.

The results are presented as the means ± standard deviation (SD) of two to three independent experiments. Statistical analysis was performed using one-way analysis of variance followed by a Dunnett’s multiple-comparison test or Student’s *t* test. A value of *P ≤ *0.05 is considered statistically significant. Data were analyzed using GraphPad Prism software.
