# Supplementary material for: FDA-Approved Amoxapine Effectively Promotes Macrophage Control of Mycobacteria by Inducing Autophagy
Source: Microbiol Spectr. 2022 Sep 21;10(5):e02509-22. doi: 10.1128/spectrum.02509-22 (PMC9602717; doi:10.1128/spectrum.02509-22)
Supplement: Supplemental file 1 — Supplemental material. Download spectrum.02509-22-s0001.pdf, PDF file, 0.6 MB [file spectrum.02509-22-s0001.pdf]

Table S1: Fifty-eight FDA-approved drugs used for the study

**Drug Name**	**Drug class**
Propafenone-HCl	Antiarrhythmic
Dihydroergotamine mesylate	Antimigraine, Vasoconstrictor, Analgesic
Haloperidol	Antipsychotic, Schizophrenia treatment
Apomorphine-HCl hemihydrate	Non selective dopamine agonist and anti - Parkinsonian
Promethazine-HCl	Anti-Allergic, Sedative
Quetiapine Fumarate	Antipsychotic
Imipramine	Antidepressant
Amoxapine	Antidepressant
Entacapone	Antiparkinsonian
Carvedilol	Antihypertensive, Congestive heart failure treatment
Gefitinib	Antineoplastic
Nimodipine	Antihypertensive, Vasodilator
Trifluoperazine-HCl	Antipsychotic, Antiemetics
Bromocriptine mesylate	Antiparkinson, Antidyskinetic
Ethacrynic acid	Diuretic
Raloxifene-HCl	Antihypocalcemic, Osteoporosis Prophylactic, Bone Density Conservation Agent
Zafirlukast	Antiasthmatic
Granisetron-HCl	Antiemetic
Aspirin	Analgesic
Mesalamine	Anti-inflammatory
Carboplatin	Antineoplastic
Danazol	Estrogen Antagonist, Endometriosis treatment
Estrone	Antineoplastic, Estrogen
Fenoprofen-Ca	Analgesic
Fenofibrate	Antilipidemic
Finasteride	Benign prostatic hypertrophy treatment
Fluorouracil	Antineoplastic
Gemfibrozil	Antilipemic
Itraconazole	Antifungal

Levonorgestrel	Contraceptive
Loratadine	Anti-Allergic, Antihistamine
Losartan-K	Antihypertensive, Antiarrhythmic
Mebendazole	Anthelmintic
Mefenamic acid	Non-steroidal anti-inflammatory, Analgesic, Antipyretic
Melphalan	A chemotherapy drug. Can be used as treatment for ovarian cancer and multiple myeloma.
Methyldopa sesquihydrate	An antihypertensive agent
Methylprednisolone	Anti-inflammatory, Antiemetic, Neuroprotective
Nabumetone	Nonsteroidal anti-inflammatory, Antineoplastic
Pantoprazole	Treats gastroesophageal reflux disease (GERD) and damage to the esophagus. Also treats high levels of acid in the stomach
Paroxetine-HCl	Antidepressant
Misoprostol	Anti-Ulcer Agent, Abortifacient Agent
Mifepristone	Contraceptive
Megestrol acetate	Contraceptive, Hormonal, Antineoplastic
Asenapine maleate	Antipsychotic
Carglumic Acid	Hyperammonaemia treatment
Carmustine	Antineoplastic
Colchicine	A natural product that is used as a medication used for gout
Desogestrel	Contraceptive
Doxapram-HCl	Respiratory stimulant
Epinastine-HCl	Antiallergic, Antihistamine, Mast cell stabilizer
Ethinyl Estradiol	Contraceptive
Etonogestrel	Hormonal contraceptive
Metaxalone	Hypnotic/Sedative, Muscle Relaxant
Mometasone Furoate	Anti-inflammatory, antiallergic
Nitisinone	hereditary tyrosinemia type 1 treatment
Orlistat	Anti-Obesity Agent
Podofilox	Antineoplastic
Trimipramine maleate	Antidepressant

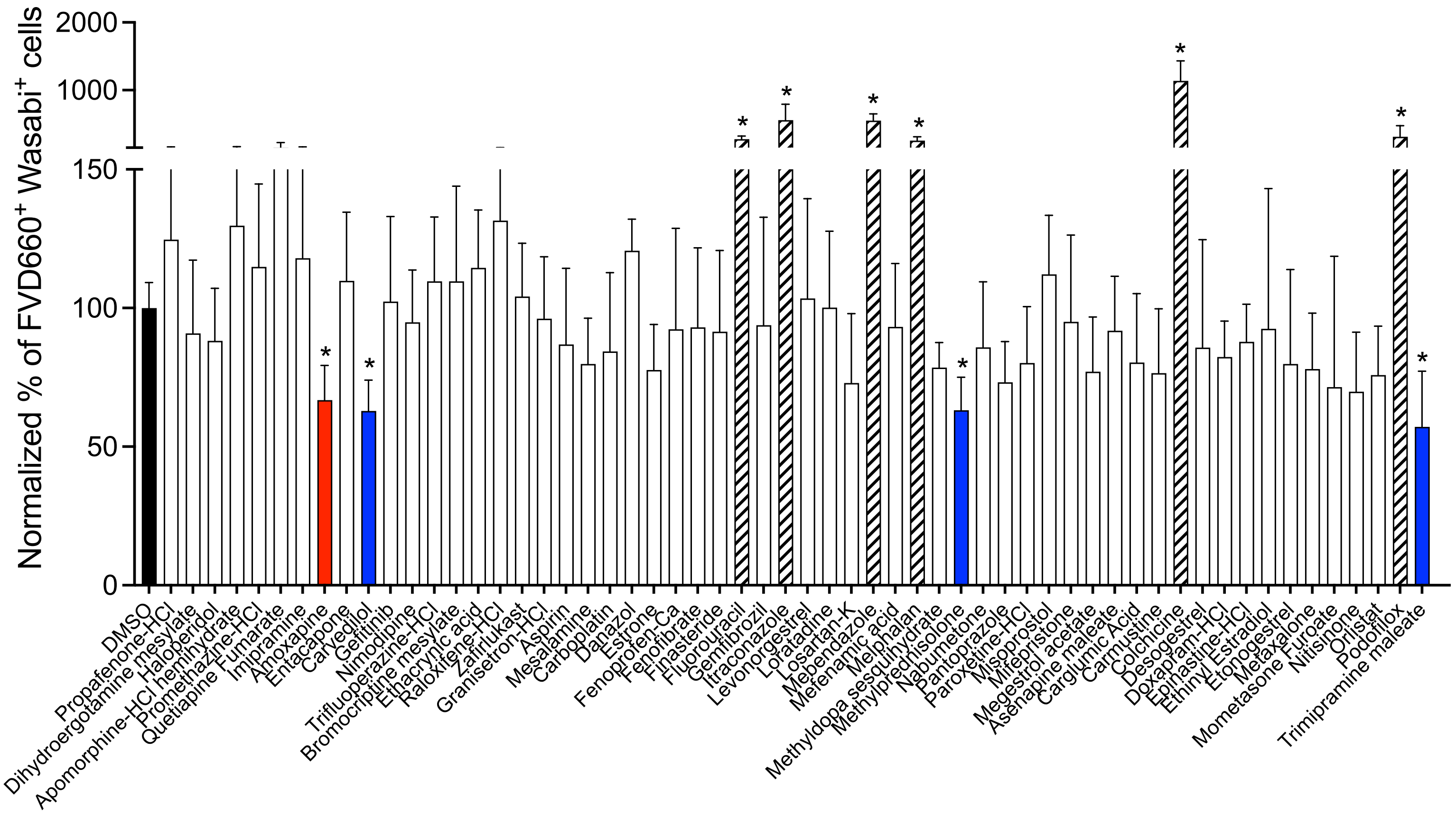

Supplementary Figure 1. The effects of 58 FDA drugs on BCG-induced macrophage cell death. RAW264.7 cells were infected with BCG-Wasabi at an MOI of 10 for 3 hours and then treated with 50 ug/ml of gentamicin for 1 hour. After washes with PBS twice, cells were incubated with 58 FDA-approved drugs along with 20 ug/ml of gentamicin for 24 hours. Macrophage cytotoxicity was evaluated by FVD660 staining and flow cytometry analysis. The data was plotted as a percentage of FVD660 and wasabi double-positive cells in drug-treated cells, normalized to DMSO treated infected RAW264.7 cells. The data represent the means ± standard deviations (SD) for two independent experiments. One-way ANOVA was used for statistical analysis to compare drug-treated to the DMSO group. *P< 0.05.

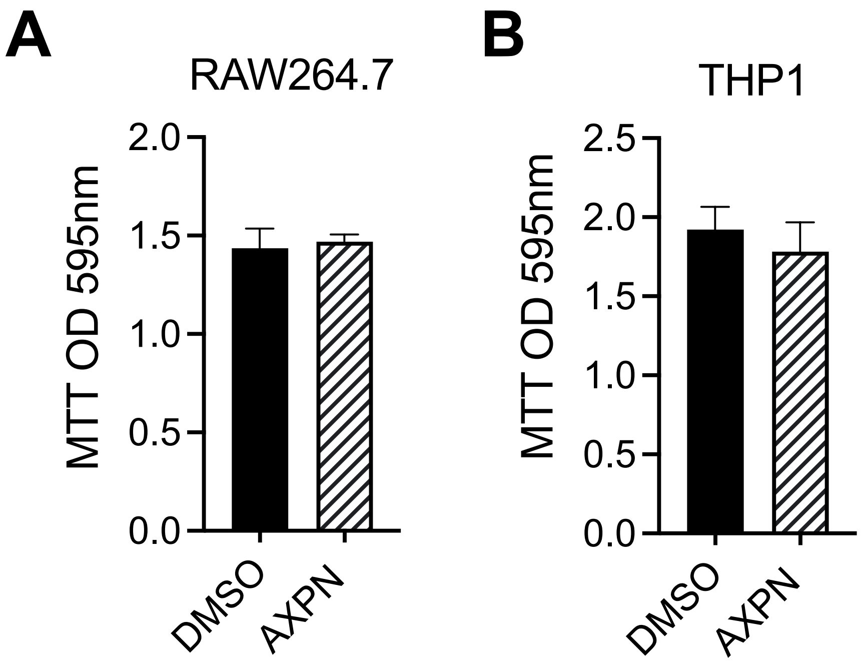

Supplementary Figure 2. Amoxapine has no significant cytotoxicity on macrophages at 10μM. (A) RAW 264.7 cells were treated with 10 μM Amoxapine for 2 days and cell viability was measured by MTT assay. (B) THP1 cells were treated with 10 μM Amoxapine for 3 days and MTT assay was used to measure cell viability. The data represent the means ± SD for two independent experiments. The student t-test was used for statistical analysis.

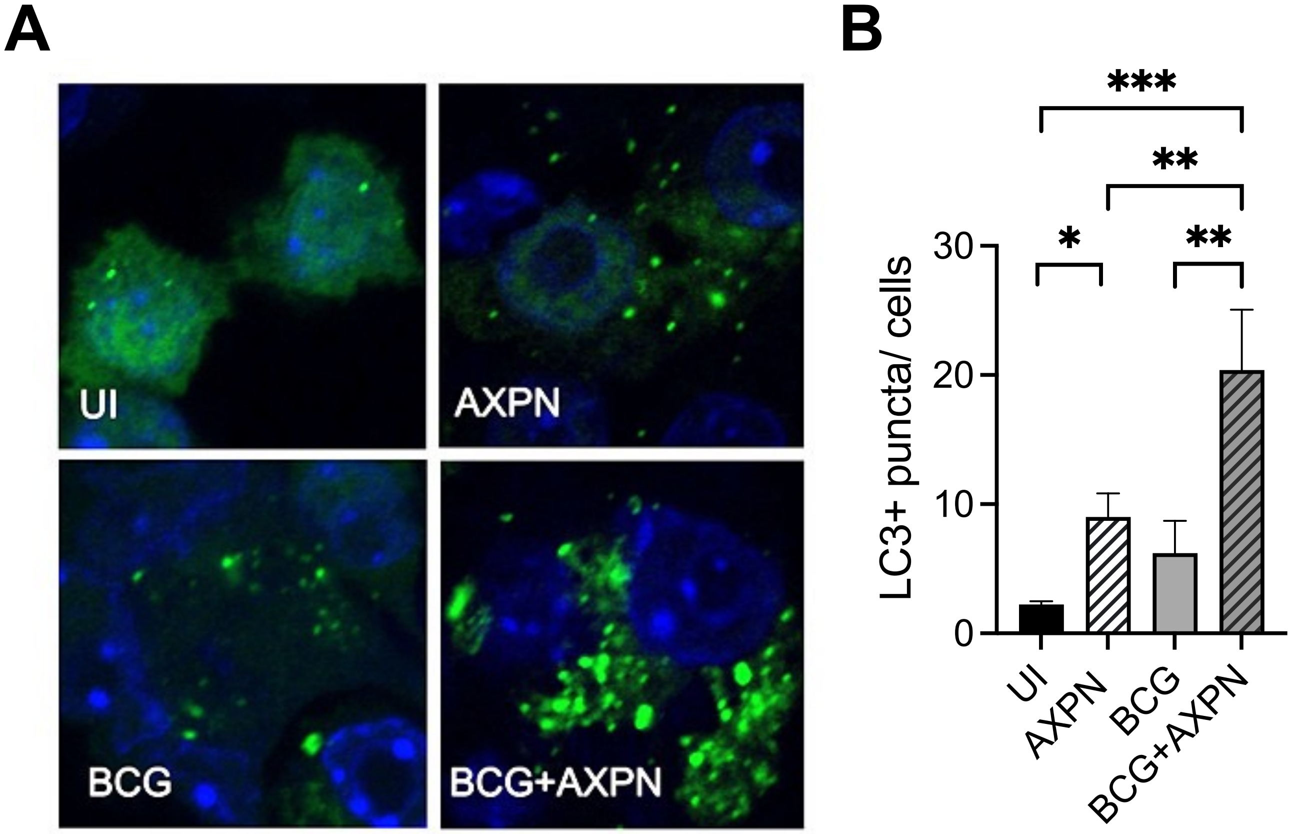

Supplementary Figure 3. Amoxapine induces autophagy in macrophages. RAW 264.7 LC3-GFP cells were infected with BCG Danish at an MOI of 10 for 3 hours and then treated with 10 µM Amoxapine for 24 hours. Cells were fixed and stained with DAPI. The confocal images were acquired by using an A1 Nikon confocal microscope with a 60X objective lens and puncta analyses were performed by NIS Elements software. One-way ANOVA with Dunnett's test was used for statistical analysis to compare drug-treated and infected groups to the untreated and uninfected (UI) control group. *P< 0.05; **P< 0.01, ***P< 0.001.

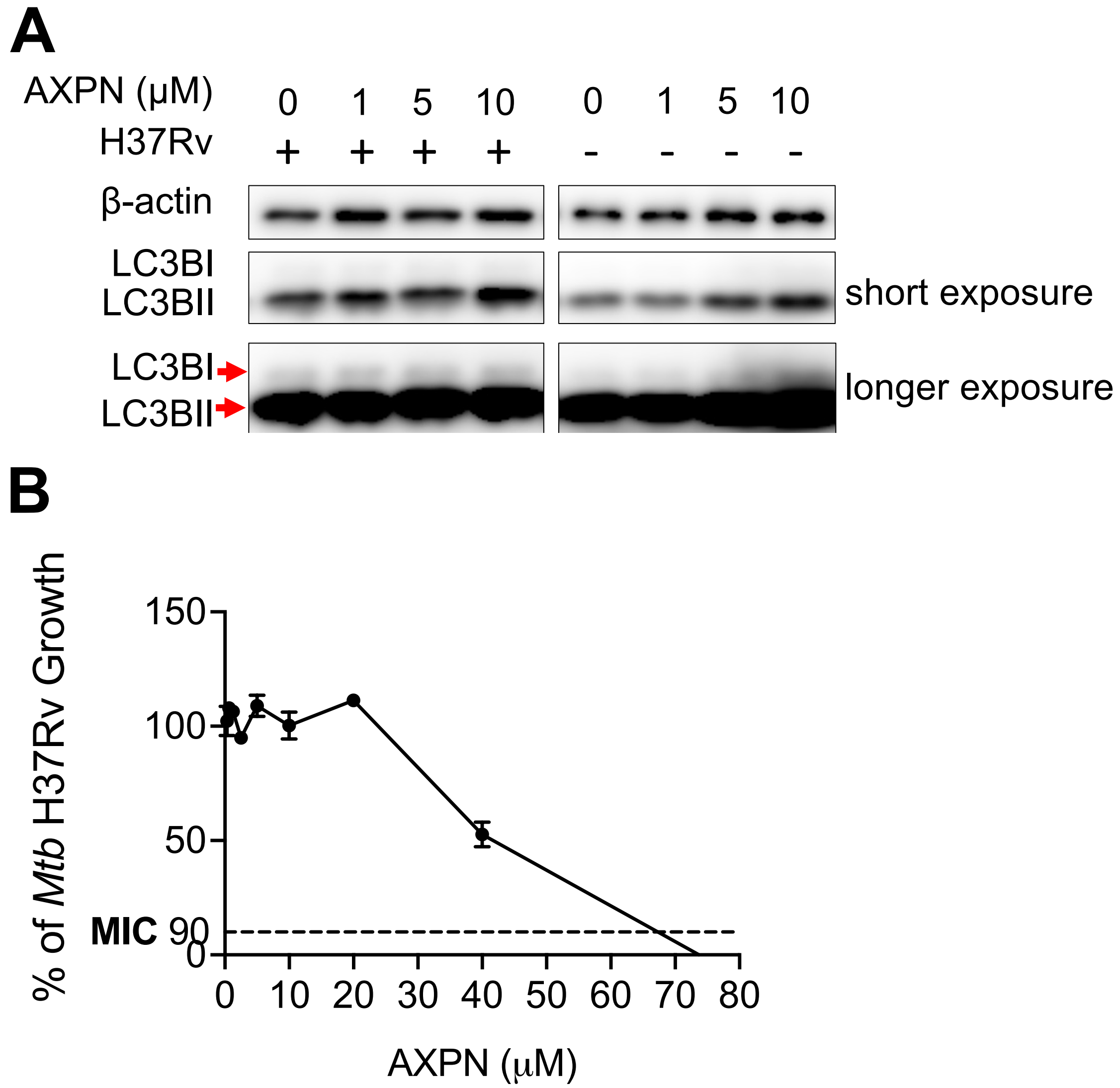

Supplementary Figure 4. (A) Amoxapine induces LC3B-II levels in primary murine bone marrow-derived macrophages. Murine BMDMs were infected with *Mtb* H37Rv at an MOI of 10 for 4 hours and treated with Amoxapine at indicated concentrations, or BMDMs were uninfected and treated with Amoxapine for 3 days. LC3B-I, LC3B-II, and actin levels in murine BMDMs were determined by western blots. Shorter and more prolonged exposures are shown. (B) Resazurin microtiter assay was used to evaluate the sensitivity of *Mtb* H37Rv to Amoxapine treatment after 5 days of incubation.

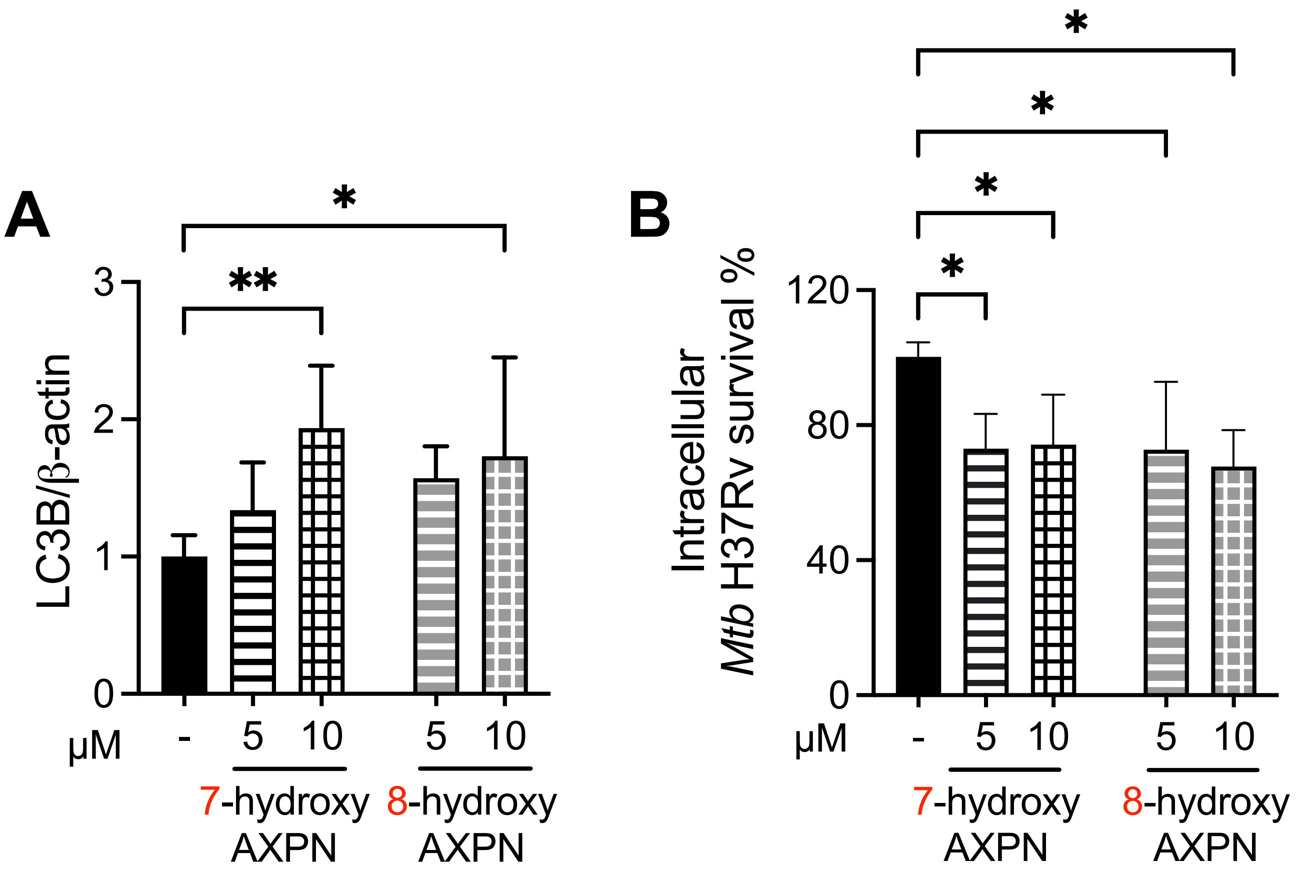

Supplementary Figure 5. Post-treatment with metabolites of Amoxapine reduces intracellular survival of *Mtb* H37Rv in THP1 cells. THP1 cells were infected with *Mtb* H37Rv at an MOI of 10 for 4 hours and then treated with 7-hydroxyamoxapine or 8-hydroxyamoxapine at concentrations of 5 and 10 μM for 2 days. (A) LC3B-II levels were determined by western blots and quantified by Image J. (B) Intracellular bacterial load was enumerated. The data represent the means ± SD for two independent experiments. One-way ANOVA with Dunnett's test was used for statistical analysis to compare drug-treated groups to the untreated control group. *P< 0.05.

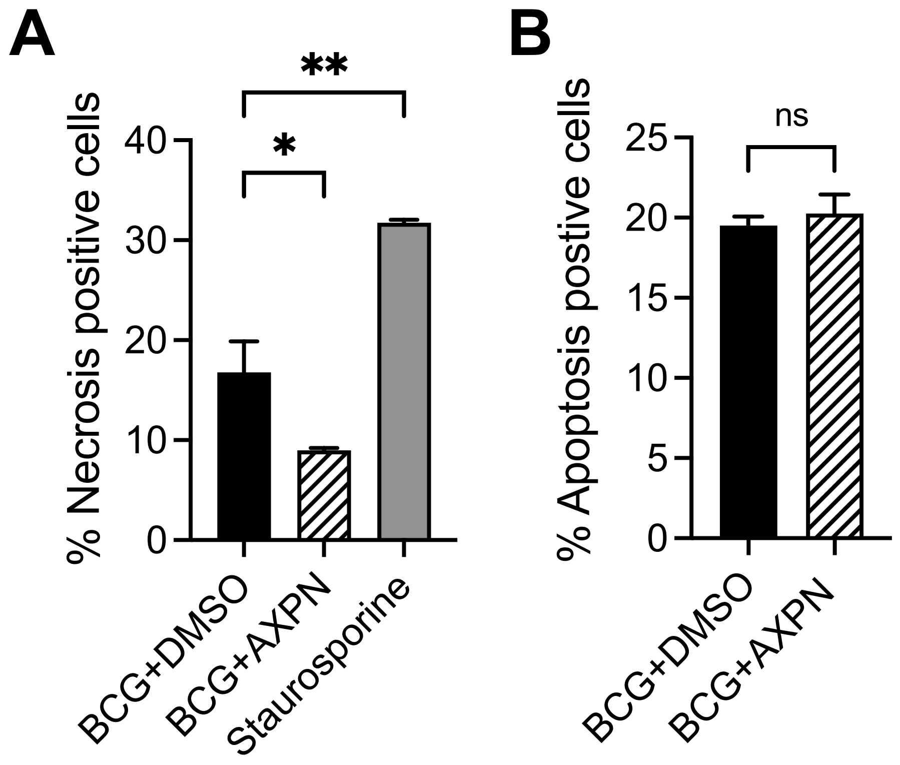

Supplementary Figure 6. Post-treatment with Amoxapine inhibits necrosis without affecting apoptosis. RAW 264.7 cells were infected with BCG-Wasabi at an MOI of 10 for 3 hours and then treated with 10 μM Amoxapine for 24 hours. Cells were stained with GFP-certified Apoptosis/Necrosis detection kit, followed by flow cytometry analysis. (A) Necrosis positive cells was plotted. (B) Apoptosis in BCG-infected macrophages were plotted. The data represent the means ± standard deviations (SD) for two independent experiments. One-way ANOVA or the student t-test was used for statistical analysis. *P< 0.05; **P< 0.01.
